# Efficacy and safety evaluation of ketogenic diet therapy in patients with glucose transporter type 1 deficiency syndrome

**DOI:** 10.3389/fnut.2026.1920521

**Published:** 2026-09-17

**Authors:** Man Wang, Li Liu, Li Shang, Dandan Huang, Qin Li, Hui Guo, Yingyan Wang, Huanling Yu

**Affiliations:** 1 Shanghai Deji Hospital, Qingdao University, Shanghai, China; 2 School of Public Health, Capital Medical University, Beijing, China; 3 Shanghai Children’s Medical Center, Shanghai Jiao Tong University School of Medicine, Shanghai, China

**Keywords:** efficacy, epilepsy, glucose transporter type 1 deficiency syndrome (GLUT1-DS), ketogenic diet, SLC2A1

## Abstract

**Objective:**

Glucose transporter type 1 deficiency syndrome (GLUT1-DS) presents with diverse clinical manifestations and varying degrees of severity. This study aims to investigate the clinical characteristics of patients with GLUT1-DS and to evaluate the efficacy and safety of ketogenic diet (KD).

**Methods:**

Clinical data were retrospectively collected from 56 patients with GLUT1-DS treated at Shanghai Deji Hospital between March 2015 and May 2024. We analyzed the initial presentations, primary clinical manifestations, cerebrospinal fluid (CSF) characteristics, and genetic testing results, as well as the efficacy and safety of ketogenic diet therapy (KDT).

**Results:**

The median age of onset was 6.0 months, and diagnosis was confirmed at a median age of 32.5 months. Epilepsy was the initial symptom in 69.6% of cases, followed by movement disorders (17.9%) and developmental delay (12.5%). CSF analysis revealed a mean glucose level of 1.80 ± 0.33 mmol/L and a CSF-to-blood glucose ratio of 0.39 ± 0.07. Genetic testing confirmed *SLC2A1* mutations in all patients, predominantly *de novo* variants (98.2%). KD yielded a median response time of 2 weeks. The respective seizure control rates at 1, 3, 6, 12, and 24 months were 83.3, 95.2, 97.6, 90.0, and 85.7%. All patients with movement disorders showed improvement, with 67.6% achieving complete resolution. Furthermore, caregiver-reported language and cognitive functions showed improvement in 39.3% of patients. The most common adverse effects of KD were mild gastrointestinal symptoms, and only one patient discontinued treatment due to intolerance.

**Conclusion:**

Our findings support the effectiveness and acceptable short-term safety of KDT for patients with GLUT1-DS. KD was associated with improved seizure control, motor function, and caregiver-reported gains in language and cognition. These findings support current recommendations favoring early diagnosis and timely initiation of treatment.

## 1 Introduction

Glucose transporter type 1 deficiency syndrome (GLUT1-DS), first characterized by De Vivo et al. in 1991 (1), is an inborn error of metabolism caused by deficiency or loss of function of the Glut1 protein which is encoded by the *SLC2A1* gene (2). This disease is marked by significant clinical heterogeneity and variable severity, with hallmark features including infantile-onset epileptic seizures, paroxysmal movement disorders, and psychomotor developmental delay (3, 4). Although GLUT1-DS is primarily associated with *SLC2A1* gene and follows an autosomal dominant inheritance pattern, most cases are sporadic and result from *de novo* variants (5). Phenotypic severity generally correlates with the specific genotype: missense variants typically result in mild-to-moderate presentations; splice site, nonsense, insertion, deletion, and exon deletion variants are frequently linked to moderate-to-severe manifestations; and whole-gene deletions lead to the most severe phenotypes (6).

According to the 2020 consensus guidelines from the International Glut1DS Study Group (7), the diagnosis of GLUT1-DS is based on three core criteria: First, clinical manifestations; Second, cerebrospinal fluid (CSF) biochemical abnormalities, defined as CSF glucose concentration <2.9 mmol/L; Third, genetic evidence, demonstrated by identification of a pathogenic *SLC2A1* variant. A definitive diagnosis can be established by meeting any of the following conditions: All three criteria (clinical features + CSF abnormalities + *SLC2A1* variant); Clinical features and genetic evidence; In cases where *SLC2A1* genetic testing is not performed or results are negative, a clinical diagnosis may be established if criteria 1 and 2 are met and exclusion criteria (e.g., mitochondrial disorders, hypoglycemia, or other metabolic diseases) are satisfied.

Ketogenic diet (KD) is a specialized medical regimen characterized by high fat, adequate protein, and low carbohydrate content, providing an alternative metabolic fuel for the developing brain in patients with GLUT1-DS. Currently, four primary KD variants are currently used: the classical ketogenic diet (CKD), the modified Atkins diet (MAD), the medium-chain triglyceride diet (MCT), and the low glycemic index treatment (LGIT). Ketogenic diet therapy (KDT) serves as an effective intervention for GLUT1-DS and should be initiated promptly following a confirmed diagnosis (8–10). In the management of this disorder, KD transcends the conventional definition of a “diet,” functioning fundamentally as a “food-as-medicine” therapeutic strategy. Although domestic case reports and multicenter studies on GLUT1-DS have emerged in recent years (11), the initiation of KD remains delayed, and systematic follow-up data regarding its efficacy and safety are currently lacking.

Therefore, we conducted a retrospective analysis of 56 patients diagnosed with GLUT1-DS at our center. This study summarizes their clinical characteristics, KDT selection, time to initial efficacy, maintenance of therapeutic response, and adverse events. Our findings aim to support early clinical recognition and individualized dietary management, thereby improving clinical outcomes and increasing dietary retention rates.

## 2 Methods

### 2.1 Study population

This retrospective study included 56 patients diagnosed with GLUT1-DS at Shanghai Deji Hospital between March 2015 and May 2024. All participants met the diagnostic criteria for GLUT1-DS based on clinical phenotypes, CSF glucose levels, and genetic testing results (7). KDTs were initiated in all patients following a confirmed diagnosis. This study was approved by the Clinical Research Ethics Committee of Shanghai Deji Hospital (Approval No. SDJH-LL-2024-003), and written informed consent was obtained from all patients and their legal guardians.

### 2.2 Clinical data collection

A standardized data collection form was developed to systematically collect patient data, including general demographics (sex, age, height, and weight), key clinical history (age at onset, initial symptoms, and age at diagnosis), symptom phenotypes (epilepsy, movement disorders, and cognitive impairment), use of anti-seizure medications (ASMs), CSF glucose and concurrent blood glucose levels, and genetic testing results. Data collection strictly adhered to ethical guidelines to ensure the reliability and clinical utility of the study.

### 2.3 KDT-related analysis

An individualized KDT protocol was developed for each patient, along with a standardized follow-up form to monitor treatment efficacy. We retrospectively analyzed the safety and efficacy of KDTs, including initiation timing, dietary ratios, treatment duration, therapeutic outcomes, and adverse events. The design of the KDT protocols adhered to three core principles: (1) maintaining a ketogenic ratio between 1:1 and 4:1 (fat + protein: carbohydrates); (2) achieving a stable blood ketone-to-glucose ratio; and (3) implementing personalized ketogenic meal plans with standardized training on recipe formulation principles, measurement metrics, and preparation methods.

Detailed KDT protocols are provided in the Supplementary Material. In brief, we used a non-fasting gradual escalation over 3–7 days to improve tolerance. CKD was the main regimen, with ratios from 2:1 to 4:1; MAD, MCT, and LGIT were reserved for selected patients based on age and tolerance. Blood ketones were checked 3–4 times daily during initiation and weekly thereafter, aiming for 1.0–5.0 mmol/L. We adjusted the diet monthly based on growth, seizure response, and side effects. Adherence was tracked through diet records, caregiver reports, and ketone levels. Nutritional follow-up included growth, micronutrients, and metabolic labs at 1, 3, 6, 12, and 24 months. Full detailed protocols on initiation, ratios, monitoring, adherence, adjustments, and follow-up are presented in the Supplementary Material.

### 2.4 KDT follow-up and efficacy assessment

The efficacy of KDT was evaluated at 1, 3, 6, 12, and 24 months after initiation, with detailed assessments of seizure frequency and electroencephalogram (EEG) improvements. Seizure outcome analysis was restricted to patients who had active seizures within the 3 months prior to KDT initiation. (1) Epilepsy outcomes: Efficacy was assessed using the Engel classification system (12), divided into four classes: Class I (completely seizure-free); Class II (≥90% reduction in seizure frequency); Class III (≥50% reduction); and Class IV (<50% reduction). Classes I, II, and III were considered effective responses, whereas Class IV and cases withdrawn from treatment for any reason were classified as ineffective. The overall response rate was calculated as [(Class I + II + III) / total cases] × 100%. EEG findings before and after KD treatment were also compared. (2) Paroxysmal movement disorders: Complete resolution or improvement of symptoms was defined as an effective response; lack of improvement was considered ineffective. (3) Language, cognition, and behavior: Improvements were assessed by parents based on expressive language ability, memory, command execution, learning capacity, and attention span. Any observed improvement was classified as effective, while no change was deemed ineffective.

### 2.5 Statistical analysis

The efficacy of KDTs in patients was evaluated based on the Engel classification criteria. Statistical analyses were performed with SPSS version 26 software. Non-normally distributed continuous variables-including age at onset, age at diagnosis, time to onset of KD efficacy (weeks), and duration of KD-are expressed as median with interquartile range [M (Q1, Q3)]. Normally distributed continuous variables, such as CSF glucose levels, blood glucose, and the CSF-to-blood glucose ratio, are presented as mean ± standard deviation (SD). Given the small sample size and the limited number of non-responders, we restricted our analysis to descriptive statistics. Multivariable regression was not attempted.

## 3 Results

### 3.1 Demographic characteristics

A total of 56 pediatric patients with GLUT1-DS were enrolled in this study, including 25 males and 31 females. The median age of disease onset was 6.0 months (IQR: 3.00–14.00), and the median age at diagnosis was 32.5 months (IQR: 13.75–60.50). The median interval between diagnosis and KD initiation was 1 month (IQR: 0.00–5.75).

### 3.2 Clinical characteristics

#### 3.2.1 Initial presenting symptoms

Seizure was the most common initial symptom, occurring in 39 patients (69.64%), followed by movement disorders in 10 patients (17.86%) and psychomotor developmental delay in 7 patients (12.50%) (shown in Table 1).

**Table 1:** Demographic and clinical characteristics of patients.

**Variable**	**Total (*n* = 56)**
Gender, *n* (%)	
Male	25 (44.64%)
Female	31 (55.36%)
Height (cm), Mean ± SD	112.48 ± 23.27
Weight (kg), Median (Q1, Q3)	19.75 (13.75, 25.0)
Age at onset (months), Median (Q1, Q3)	6.0 (3.0, 14.0)
Age at diagnosis (months), Median (Q1, Q3)	32.5 (13.75, 60.5)
Blood glucose (mmol/L), Mean ± SD	4.69 ± 0.65
CSF glucose (mmol/L), Mean ± SD	1.80 ± 0.33
CSF/blood glucose ratio, Mean ± SD	0.39 ± 0.07
Symptoms at onset, *n* (%)	
Epilepsy	39 (69.64%)
Psychomotor developmental delay	7 (12.50%)
Movement disorders	10 (17.86%)
Clinical manifestations, *n* (%)	
Epilepsy	50 (89.28%)
Psychomotor developmental delay	49 (87.50%)
Movement disorders	37 (66.07%)
Genetic mutation types, *n* (%)	
Missense mutation	29 (51.79%)
Deletion mutation	3 (5.36%)
Nonsense mutation	6 (10.71%)
Frameshift mutation	13 (23.21%)
Chromosomal heterozygous deletion	5 (8.93%)

#### 3.2.2 Clinical manifestations

Epileptic seizures were observed in 50 patients (89.28%). Seizure types included focal epilepsy (*n* = 10), generalized epilepsy (*n* = 37; comprising generalized tonic–clonic [*n* = 24], atypical absence [*n* = 9], atonic [*n* = 3], and clonic [*n* = 1] seizures), focal to bilateral tonic–clonic seizures (*n* = 2), and unknown onset (*n* = 1). Five patients presented with two or more seizure types. Notably, six patients remained seizure-free and another two patients had no active seizures in the 3 months before KDT initiation, and the remaining 42 patients were evaluated at each time point.

Anti-seizure medications, prior to KD initiation, were used in 39 patients. The most commonly used ASMs were levetiracetam (*n* = 15, 38.46%), valproate (*n* = 13, 33.33%), and oxcarbazepine (*n* = 10, 25.64%). Monotherapy was administered to 22 patients (56.41%), while 17 patients (43.59%) received polytherapy.

Movement disorders were observed in 37 patients (66.07%), primarily manifesting as paroxysmal limb weakness (*n* = 18), paroxysmal ataxia (*n* = 16), paroxysmal head-eye movements (*n* = 7), and abnormal saccadic gaze (*n* = 5). Psychomotor developmental delay of varying degrees was present in 49 patients (87.50%), including global developmental delay (*n* = 41), isolated language delay (*n* = 5), and isolated motor delay (*n* = 3).

#### 3.2.3 Laboratory investigations

1. CSF and blood glucose analysis: Lumbar puncture (LP) with concurrent blood glucose measurement was performed in 42 patients. The mean CSF glucose level was 1.80 ± 0.33 mmol/L, and CSF-to-blood glucose ratio was 0.39 ± 0.07. Specifically, 39 patients had a CSF glucose level <2.2 mmol/L, while 3 patients had levels ≥2.2 mmol/L (2.24, 2.39, and 2.40 mmol/L, respectively), with the lowest recorded value 0.92 mmol/L. Regarding the CSF-to-blood glucose ratio, 23 patients (54.76%) had a ratio <0.4, 17 patients (40.48%) had a ratio between 0.4 and 0.5, and 2 patients had a ratio >0.5 (0.52 and 0.525), with corresponding CSF/blood glucose values of 1.92/3.71 mmol/L and 2.1/4.0 mmol/L, respectively. Fourteen patients did not undergo LP. For these cases, as well as those with atypical CSF findings, the diagnosis was definitively established through genetic testing.
2. Pre-KDT MRI and EEG findings: EEG prior to KDT initiation was conducted in 55 patients. Interictal epileptiform discharges indicating abnormal EEG findings were observed in 40 patients (72.72%), while 15 patients had normal EEG results. Magnetic resonance imaging (MRI) was performed in 33 patients. Among them, 14 scans (42.42%) were normal, and 19 patients (57.57%) exhibited non-specific abnormalities, such as delayed myelination and ventriculomegaly.
3. Genetic testing results: Whole-exome sequencing (WES) was performed in all 56 patients. The identified *SLC2A1* mutation types included missense mutations (*n* = 29, 51.79%), frameshift mutations (*n* = 13, 23.21%), nonsense mutations (*n* = 6, 10.71%), deletion mutations (*n* = 3, 5.36%), and heterozygous chromosomal deletions (*n* = 5, 8.93%). Among the 56 identified variants, 55 were *de novo*, and one was maternally inherited.

### 3.3 KDT protocols, efficacy, and adverse events

#### 3.3.1 KDT protocols

Thirty-eight patients (67.86%) received CKD with ratios ranging from 1:1 to 3:1; 4 patients (7.14%) received MAD, 1 MCT and 1 LGIT. Additionally, 11 patients (19.64%) transitioned from CKD to MAD, and 1 patient (1.79%) switched from LGIT to MAD during the treatment period (see Table 2). Regarding blood glucose and ketone levels during KDT, long-term blood ketone levels were maintained above 1.0 mmol/L in all cases. Notably, among patients achieving Engel Class I seizure control, blood ketone levels were consistently maintained >2.0 mmol/L.

**Table 2:** KDT follow-up and adverse events.

**Variable**	**Total (*n* = 56)**
Time from onset to KDT initiation (months), Median (Q1, Q3)	24.0 (10.0, 57.5)
Time from diagnosis to KDT initiation (months), Median (Q1, Q3)	1.0 (0.0, 5.75)
Time to KDT efficacy (weeks), Median (Q1, Q3)	2.0 (1.0, 4.0)
KD type, *n* (%)	
CKD	38 (67.86%)
MCT	4 (7.14%)
MAD	1 (1.79%)
LGIT	1 (1.79%)
CKD → MAD	11 (19.64%)
LGIT→MAD	1 (1.79%)
Duration of KDT (months), Median (Q1, Q3)	33.0 (13.75, 59.25)
Number of patients with side effects	n/total (%)
1 month	13/56 (23.21%)
3 months	9/56 (16.07%)
6 months	6/55 (10.90%)
12 months	7/50 (14.00%)
24 months	3/33 (9.09%)

#### 3.3.2 Efficacy assessment

Based on a comprehensive analysis of improvements in seizures, movement disorders, and cognitive function, the median time to onset of KD efficacy was 2 weeks (IQR: 1–4), and the median duration of treatment was 33 months (IQR: 13.75–59.25).

1. Seizure outcomes: 42 patients (84% of the 50 patients with epilepsy) experienced seizures prior to KD initiation. At 1 month after treatment, 35 patients (83.33%) showed a positive response: 27 patients achieved Engel Class I, 4 Class II, and 4 Class III; 7 patients were non-responders (Engel Class IV), with 3 exhibiting increased seizure frequency. At 3 months, the number of responders increased to 40 (95.23%), including 35 in Class I, 3 in Class II, 2 in Class III. The proportion of effective responders was 97.56% (40/41) at 6 months and 90% (36/40) at 12 months. At 24 months, among the 28 patients still on treatment, 24 (85.71%) were responders (Table 3).
2. EEG Findings pre- and post-KD: Pre-KD EEG was performed in 55 patients, of whom 15 (27.27%) showed normal results and 40 (72.73%) showed abnormal findings. Follow-up EEGs showed normalization in 12 of 22 patients (54.54%) at 3 months, 6 of 10 patients (60.00%) at 6 months, and 7 of 15 patients (46.67%) at 12 months. However, at 24 months, only 1 of 13 patients (7.69%) had a normal EEG, while 12 of 13 (92.31%) remained abnormal (Table 4). Of note, the actual number of follow-up EEGs was lower than the total cohort size, as many patients with stable conditions did not undergo routine EEG monitoring.
3. Changes in paroxysmal movement disorders: Prior to treatment, 37 patients (66.07%) exhibited varying degrees of paroxysmal movement disorders. Following KDT, all patients showed improvement: symptoms completely resolved in 25 patients (67.57%) and partially improved in 12 patients (32.43%).
4. Changes in language and cognitive function: Before treatment, 49 patients (87.50%) presented with varying degrees of language and cognitive impairment based on caregiver reports. Following KDT, caregivers of all children reported improvements in language and cognition. Notably, families of 22 patients (22/56, 39.29%,) reported that their children’s development had reached the baseline level of typically developing peers.

**Table 3:** Evaluation of seizure control by Engel classification in patients with epilepsy.

Engel class	After KDT				
	1 month (*n* = 42)	3 months (*n* = 42)	6 months (*n* = 41)	12 months (*n* = 40)	24 months (*n* = 28)
Class I	27(64.29%)	35(83.33%)	36(87.80%)	29(72.50%)	20(71.43%)
Class II	4(9.52%)	3(7.14%)	0(0.00)	3(7.50%)	0(0.00)
Class III	4(9.52%)	2(4.76%)	4(9.76%)	4(10.00%)	4(14.29%)
Class IV	7(16.67%)	2(4.76%)	1(2.44%)	4(10.00%)	4(14.29%)

KDT: ketogenic diet therapy.

**Table 4:** Summary of EEG findings at different time points.

Follow-up period	Number of participants tested
Before KDT, *n*	55
Normal	15(27.27%)
Abnormal	40(72.73%)
After KDT	
3 months, *n* (%)	22
Normal	12 (54.54%)
Abnormal	10 (45.46%)
6 months, n (%)	10
Normal	6 (60.00%)
Abnormal	4 (40.00%)
12 months, n	15
Normal	7 (46.67%)
Abnormal	8 (53.33%)
24 months, n	13
Normal	1 (7.69%)
Abnormal	12 (92.31%)

KDT: ketogenic diet therapy.

#### 3.3.3 Assessment of adverse events

Adverse events were most frequent during the first month of KDT (13/56, 23.21%) and gradually decreased over time (9/56 at 3 months; 6/55 at 6 months; 7/50 at 12 months; and 4/33 at 24 months). The most common complications were gastrointestinal symptoms, including vomiting, diarrhea, constipation, abdominal pain, and poor appetite. Other reported events included transient food refusal, decreased immunity, hypercholesterolemia, hyperuricemia, irritability, and malnutrition. Throughout the study, no patients experienced severe complications such as persistent hypoglycemia, metabolic acidosis, liver dysfunction, electrolyte disturbances, or urolithiasis.

### 3.4 Participant flow and follow-up

All 56 patients were included in the baseline analysis. The number of patients with available data varied across time points and between analyses. For adverse events (Table 2), the denominator fell from 56 at 1 month to 33 at 24 months. This was mainly because some patients had not yet reached the 24-month time point by the data cutoff, while others had stopped the diet earlier due to intolerance or poor adherence. For seizure outcomes (Table 3), the analysis started with 42 patients who had active seizures before KDT. The denominator dropped to 28 by 24 months, largely because patients who became seizure-free (Engel Class I) and completed their scheduled visits were not routinely followed further, which reflected our standard clinical practice. For EEG (Table 4), follow-up scans were done on clinical indication rather than a fixed research protocol. Patients who were clinically stable were simply less likely to have repeat EEGs, which made the later time-point samples smaller and more selected.

## 4 Discussion

GLUT1-DS is a rare neurometabolic disorder caused by defects in the *SLC2A1* gene, clinically characterized by the hallmark triad of epilepsy, paroxysmal movement disorders, and psychomotor developmental delay (13). KD can supply ketone bodies as an alternative cerebral fuel for impaired glucose transport and is widely recognized as the first-line treatment for this disease. In the present study, we conducted systematic follow-ups on 56 pediatric patients with GLUT1-DS undergoing KDT, further confirming its substantial efficacy in seizure control, as well as the improvement in motor disorders and cognitive function.

Approximately 90% of patients with GLUT1-DS present with epilepsy, typically manifesting around a median age of 8 months and rarely during the neonatal period (14, 15). In our cohort of 56 patients, 39 (69.64%) had seizures as the initial symptom, with a median onset age of 6.0 months (range: starting at 1 month), consistent with the characteristic early-infantile presentation. The median age at diagnosis was 32.5 months, with the longest diagnostic delay reaching 13.3 years. Seizure semiology in GLUT1-DS is highly variable, while a minority of patients presenting with phenotypes like idiopathic generalized epilepsy responded to ASMs (16). Among the 50 patients with seizures in our cohort, generalized tonic–clonic and atypical absence seizures were predominant, yet focal epilepsy also accounted for a substantial proportion (28.60%), underscoring that focal onset remains a critical diagnostic clue despite typical generalized symptom. Notably, only 8 patients (16%) achieved effective control with ASMs prior to KD, and 6 experienced increased seizure frequency upon ASM withdrawal during KD, suggesting only partial pharmacological efficacy.

Ketogenic diet enables most patients to achieve seizure freedom or reduction, discontinue ASMs, and normalize EEG findings (17). However, a subset of patients remained refractory to KDT and failed to achieve seizure control. The efficacy of KDT showed no correlation with seizure type, clinical phenotype, or genetic characteristics, reflecting the disease’s complexity. Although ASMs may benefit some patients, no single drug has demonstrated a clear advantage (18). The persistence of seizures in a subset of patients despite KD indicates that seizure can remain refractory in GLUT1-DS, warranting further investigation into the underlying mechanisms. We also monitored EEG evolution over time. Pre-KDT, 72.73% (40/55) of EEGs were abnormal. This proportion decreased to 45.45% (10/22) at 3 months and 40% (4/10) at 6 months, but notably rose to 92.31% (12/13) at 24 months. However, since many clinically seizure-free children did not undergo routine follow-up EEGs, this trend might be influenced by reduced sample size and selection bias. Beyond this, several other factors might contribute to the rising abnormality rate. First, we noticed that adherence often dropped with age. Whether this caused unstable ketosis or contributed to EEG changes is uncertain and needs further study. Second, ASMs were withdrawn in many patients during follow-up. Whether this withdrawal uncovered EEG abnormalities was possible, but we cannot confirm this with our data. Third, the small and non-consecutive sample sizes at each time point introduced a selection bias, as patients with persistent clinical concerns were more likely to undergo repeated EEG testing, while those with sustained normalization and stable outcomes were less frequently re-evaluated. Lastly, our analysis was mostly descriptive. We did not perform formal tests for factors linked to treatment response, because of the small number of non-responders. Collectively, these factors highlight the need for standardized, protocol driven EEG monitoring in future prospective studies to better assess the long-term electrophysiological impacts of KDT.

Patients with GLUT1-DS may present with various movement disorders. Typical manifestations include paroxysmal ataxia, dystonia, chorea, and intention tremor, while atypical features encompass ocular motor abnormalities and paralysis (19, 20). Literature reports indicate that approximately 32% of patients exhibit abnormal eye movements in early infancy; notably, these can precede seizure onset and serve as an early clinical clue for diagnosis (21). However, in our cohort, only 5 patients (8.93%) reported such symptoms, a rate significantly lower than reported in the literature. This discrepancy may be attributed to parental oversight during the early stages of the disease, so clinicians should actively inquire about this symptom as it is frequently overlooked by both families and healthcare providers. Among the 56 patients, 10 (17.86%) presented with movement disorders as the initial symptom, and 37 (66.07%) exhibited varying degrees of motor impairment or dystonia before treatment. Following KDT, all patients showed varying degrees of improvement: symptoms completely resolved in 67.56% (25/37) and partially improved in the remaining 32.43% (12/37). Notably, these assessments were based on caregiver reports, highlighting the necessity of employing standardized developmental scales for more rigorous evaluation in future studies.

Cerebrospinal fluid glucose is a key diagnostic marker for GLUT1-DS. LP should ideally be performed after a 4–6 h fast, before breakfast (22). Absolute CSF glucose reduction is more valuable than decreased CSF-to-blood glucose ratio (22). About 90% of patients have glucose levels below 2.5 mmol/L (23), and a ratio below 0.4 is a useful indicator in most cases (24). In our cohort, 42 of 56 patients (75.0%) underwent concurrent testing. Mean CSF glucose was 1.85 ± 0.13 mmol/L, and the CSF-to-blood ratio was 0.38 ± 0.07. Specifically, 39 patients had CSF glucose < 2.2 mmol/L, while 3 had levels ≥ 2.2 mmol/L. For the ratio, 23 patients were <0.4, 17 were between 0.4–0.5, and 2 patients had a ratio > 0.5. The earliest diagnosis among these 42 patients was at 2.3 months, highlighting the need for early standardized CSF analysis in patients with unexplained early-onset refractory epilepsy, paroxysmal movement disorders, or developmental delay. Assessing Erythrocyte membrane GLUT1 levels by flow cytometry show good sensitivity and specificity for diagnosing GLUT1-DS (25). This method is rapid, cost-effective and non-invasive, which make it an adjunctive tool particularly for families reluctant to undergo lumbar puncture.

Genetic testing serves as a crucial method for establishing a definitive diagnosis. Currently, *SLC2A1* is recognized as the primary pathogenic gene associated with GLUT1-DS. Variants are predominantly *de novo*, resulting in mostly sporadic cases inherited in an autosomal dominant pattern (26). Among the 56 patients in our cohort, 55 (98.21%) carried *de novo* mutations, while the remaining one inherited the variant maternally. In GLUT1-DS, approximately 90% of variants are nucleotide substitutions within the *SLC2A1* gene, while rarer cases involve large deletions or duplications that may encompass the entire gene. (27). Consistent with this, 51 cases (91.07%) in our study carried nucleotide substitutions, and 5 (8.93%) had chromosomal deletions. GLUT1-DS shows significant phenotypic heterogeneity: patients with identical genotypes may present with markedly different clinical features. Although pathogenic variants correlate with disease severity, no association has been found between genotype and response to KD (6). For patients with atypical clinical presentations, early genetic testing facilitates a definitive diagnosis. Furthermore, prompt molecular confirmation of the *SLC2A1* gene combined with the early initiation of KD is crucial for improving cognitive outcomes in patients with GLUT1-DS.

Ketogenic diet therapy is the first-line treatment for GLUT1-DS, improving clinical symptoms and development. KDTs should start immediately after diagnosis. The CKD is preferred for infants and preschool children to achieve better ketosis, while MAD is more suitable for school-aged children, adolescents, and adults to improve tolerance (7). LGIT may be used for maintenance in adulthood. The need for lifelong KDTs remains unclear (15, 28). Seizures are controlled within days to weeks after starting KDT, while motor and cognitive improvements usually occur over months (13). Among our 56 patients, KDT regimens were: CKD (67.86%), MAD (7.14%), MCT (1.79%), and LGIT (1.79%). During treatment, 11 patients switched from CKD to MAD, and one from LGIT to MAD, indicating that long-term tolerance and adherence to KDT are optimal. MAD serves as a viable alternative when CKD is poorly tolerated. Despite regimen diversity, the overall high response rates suggest that maintaining adequate ketosis and tolerability may be more critical in practice. All 56 patients started KDT immediately after diagnosis, and 54 derived clinical benefit. Responders tended to have a shorter disease duration before treatment, but we could not draw firm conclusions from so few non-responders. Larger studies are needed to clarify whether earlier treatment improves outcomes. A previous study reported seven KD-resistant cases, six of which had a CSF-to-blood glucose ratio >0.45 (up to 0.64) (28). In our study, the sole non-responder with intolerable side effects had a ratio of 0.44. Whether this biomarker correlates with treatment resistance warrants larger-scale investigation.

Early adverse events of KDTs were primarily gastrointestinal (diarrhea, vomiting, abdominal pain), later shifting to constipation, hyperlipidemia, and mood fluctuations (29). Persistent abdominal pain and constipation require probiotics, dietary fiber, increased fluid, and appropriate supplementation of sugar-free multivitamins. Long-term KDTs may increase the risks of fractures and osteoporosis (30). In our center, patients received vitamin D3, calcium, minerals, and multivitamins, and no fracture was observed. We recommend assessing bone age, 25(OH)D, and PTH every 6–12 months to monitor growth. Our safety findings were based on observation during the study period; however, long-term safety beyond 24 months cannot be determined from this dataset.

Despite the meaningful findings, this study has several limitations. First, as a retrospective study, it is subject to selection and information bias. Second, the follow-up duration was relatively short for some patients, which may affect a comprehensive assessment of long-term efficacy and safety. Additionally, the evaluation of language and cognitive abilities lacked standardized objective scales, therefore the cognitive benefits reported in this study should be interpreted as caregiver-reported outcomes rather than objectively measured endpoints. Future studies should employ prospective cohort designs to validate efficacy prediction models and explore individualized KDT regimens, aiming to maximize therapeutic benefits while reducing adverse effects.

In conclusion, this study systematically evaluates the efficacy and safety of KDTs in children with GLUT1-DS, supporting the current clinical practice of early initiation, sustained implementation, and multidisciplinary management of KD therapy in patients with GLUT1-DS. Future research should focus on optimizing ketogenic formulas to improve adherence, and exploring emerging therapies such as small-molecule GLUT1 enhancers or gene therapy to comprehensively improve the long-term prognosis of children with GLUT1-DS.

## Data Availability

The raw data supporting the conclusions of this article will be made available by the authors, without undue reservation.
