# Supplementary material for: Efficacy and safety evaluation of ketogenic diet therapy in patients with glucose transporter type 1 deficiency syndrome
Source: Front Nutr. 2026 Sep 17;13:1920521. doi: 10.3389/fnut.2026.1920521 (PMC13627066; doi:10.3389/fnut.2026.1920521)
Supplement: Supplementary file 1 [file Supplementary_file_1.docx]

**Detailed KDT Protocol**

**1. Diet initiation**

We assessed dietary habits and family food preferences of each patient before starting, then tailored the KDT plan accordingly. All patients followed a non‑fasting gradual escalation protocol: 1/3 to 1/2 of total daily calories on day 1, 1/2 on day 2, and full calories on day 3. We monitored closely for hypoglycemia, dehydration, and electrolyte disturbances throughout. The initiation period ranged from 3 to 7 days, adjusted individually by age, weight, and tolerance.

Classic ketogenic diet (CKD): No fasting was required on day 1. Children and adolescents started with a low‑carbohydrate transition, while infants received formula feeds. After ketosis was achieved, we restricted daily carbohydrate intake to 10-15 g. For children under 3 years old, we extended the initiation to 5-7 days and slowed the dietary progression to reduce gastrointestinal intolerance. Patients aged 3 years or older typically reached target ketosis within 3-4 days.

Modified Atkins diet (MAD): We used a gentle initiation with a strict carbohydrate limit of ≤ 20 g/day from day 1, with no restrictions on total calories or protein. Fat intake was allowed ad libitum. Ketosis was usually achieved within 2-3 days.

Medium‑chain triglyceride diet (MCT): MCT oil provided 50% of total fat intake on day 1, with the remainder from long‑chain triglycerides. MCT gradually replaced long‑chain fats while carbohydrate intake was tightly controlled. Initiation was completed within 3 days. Some patients later increased MCT intake during cold weather when ketone levels dropped.

Low glycemic index treatment (LGIT): This approach did not require rapid carbohydrate restriction. We simply removed high‑glycemic index foods such as refined rice, white flour, and sucrose, and substituted them with low‑glycemic index alternatives. Daily carbohydrate intake was reduced gradually, reaching target levels within 7 days.

**2. Standardized dietary ratios**

We calculated daily calorie requirements for each patient based on actual body weight, age, physical activity, and growth needs (children: 90-120 kcal/kg/day). All four dietary regimens were standardized, with CKD as the main intervention. The ketogenic ratio ranged from 2:1 to 4:1, adjusted to maintain blood ketones >2.0 mmol/L. Families were trained to use meal‑planning software.

CKD: Fat: (protein + carbohydrates) ratio of 2:1 to 4:1. Daily fat contributed 82-90% of total calories, protein 6-12%, and carbohydrates ≤ 6%. Protein intake was set at 1.0-1.2 g/kg/day to meet baseline growth requirements. Carbohydrates were strictly limited to 10-20 g/day, with all added sugars and refined carbohydrates excluded.

MAD: No fixed calorie ratio. The key restriction was carbohydrates ≤ 20 g/day. Fat contributed about 65% of calories, protein 20-25%. Total food intake was flexible to improve tolerance and adherence.

MCT: Fat provided about 73% of total calories, of which MCT oil accounted for 30-60% and long‑chain triglycerides the remainder. Carbohydrates contributed 17%, protein 1.2-1.5 g/kg/day. This regimen allowed more carbohydrate than CKD and was generally better tolerated.

LGIT: Carbohydrates provided about 10% of daily calories, with total intake kept at 40-60 g/day, all from low‑glycemic index foods (GI < 50). Fat contributed 60%, protein 30%. This was the least restrictive regimen.

**3. Ketosis monitoring**

We monitored ketosis primarily via blood ketone measurements, supplemented by urinary ketone testing.

Initiation phase (days 1-5): Nurses checked capillary blood ketones and glucose four times daily (03:00, 09:00, 15:00, 21:00). Target blood ketone range was 2.0-5.0 mmol/L. Urinary ketones were checked once daily, with positive readings (+++ to ++++) considered effective. If blood ketones remained <1.0 mmol/L for three consecutive days, we adjusted the diet by increasing fat and reducing carbohydrate intake.

Stable treatment phase: After target ketosis and clinical stability were achieved, we switched to random checks of blood ketone and glucose weekly or 2-3 times monthly, aiming to maintain blood ketones at 1.0-5.0 mmol/L.

Long‑term follow‑up: We checked blood ketones and fasting glucose every 3 months to assess ketosis stability and screen for hypoglycemia or hyperketosis.

**4. Adherence management and criteria**

We used a standardized system to monitor and document dietary adherence throughout the study.

Interventions to promote adherence: Families received uniform training from clinical dietitians and neurologists on dietary restrictions, food ratios, snack rules, and how to manage side effects. We provided standardized meal plans and counselled against high‑carbohydrate foods. Regular follow‑up visits and phone calls helped address problems promptly.

Adherence classification: Good adherence: Strictly followed the assigned diet, no forbidden foods, blood ketones remained within target range, no dietary interruptions. Partial adherence: Occasional minor deviations (≤ 3 times per month), blood ketones largely within target, no need for protocol changes. Poor adherence: Frequent deviations, unauthorized changes to the diet, or discontinuation, with persistently low blood ketones.

**5. Principles for dietary adjustment**

We adjusted the diet individually based on age, weight, clinical response, tolerance, and nutritional parameters.

Basic adjustments: Weight and height were recorded monthly, and daily calorie and protein intakes were updated according to growth, maintaining an appropriate ketogenic ratio.

Response‑related adjustments: If seizures or other symptoms did not improve and blood ketones remained low after 3 months on CKD, we increased the fat ratio to 3:1 or 4:1, provided the patient tolerated it. If adverse effects such as severe gastrointestinal symptoms or hyperlipidemia occurred, we reduced the fat proportion and adjusted carbohydrate and protein accordingly.

Regimen switching: Patients intolerant to CKD (e.g., severe vomiting, diarrhea, malnutrition) could be transitioned to MAD over at least 1 week to maintain ketosis stability during the switch.

Weaning: For patients who were seizure‑free for over 2 years with normalized EEG, we tapered the diet gradually, reducing the ketogenic ratio by 0.5:1 per year until regular food was resumed, to avoid rebound.

**6. Standardized nutritional follow‑up**

We followed a tiered schedule covering efficacy, nutrition, growth, and complications.

Short‑term (1-3 months): Monthly clinic or phone visits were conducted to assess seizure frequency, mental state, and gastrointestinal tolerance. Routine blood tests, liver/kidney function, electrolytes, lipids, glucose, and ketones were checked, and any abnormalities were corrected promptly.

Medium‑term (3-12 months): Patients were followed up every 3 months to evaluate micronutrients and vitamins (D, calcium, magnesium) and targeted supplementation was provided for any deficient nutrients. Cognitive and motor development were also reported.

Long‑term (>1 year): Follow‑up assessments were conducted every 6 months to monitor height, weight, and head circumference for comparison with age‑matched norms. EEG was repeated to assess long‑term seizure control. Dietary adherence and long‑term nutritional complications were tracked, and the diet was adjusted accordingly.

All the procedures described above: initiation protocols, dietary ratios, monitoring schedules, follow‑up plans, and adjustment criteria, were uniformly applied across the entire cohort. CKD was the primary intervention and strictly followed these specifications. The other three regimens (MAD, MCT, LGIT) also had clearly defined, replicable protocols. This ensures the transparency and reproducibility of our ketogenic diet therapy.
